# Use of Neural Machine Translation Software for Patients With Limited English Proficiency to Assess Postoperative Pain and Nausea

**DOI:** 10.1001/jamanetworkopen.2022.1485

**Published:** 2022-03-08

**Authors:** Ravish Kapoor, German Corrales, Manuel P. Flores, Lei Feng, Juan P. Cata

**Affiliations:** 1 Department of Anesthesiology and Perioperative Medicine, The University of Texas MD Anderson Cancer Center, , Houston; 2 Anesthesiology and Surgical Oncology Research Group, Houston, Texas; 3 Department of Biostatistics, The University of Texas MD Anderson Cancer Center, Houston

## Abstract

This cohort study investigates the use of neural machine translation software to assess postoperative pain and nausea among patients with limited English proficiency.

## Introduction

The association of language barriers with health care disparities is well described.^1^ Postoperative translation services can become overstretched when acutely needed to address patient needs.

Google Translate conversation mode is a multilingual neural machine translation service offering a free electronic interface available on mobile devices, instantly translating spoken words into 70 languages. We hypothesized that the application’s translate conversation mode may facilitate assessment of postoperative pain and nausea.

## Methods

Institutional review board approval was granted for this cohort study by MD Anderson Cancer Center. This study followed the Strengthening the Reporting of Observational Studies in Epidemiology (STROBE) reporting guideline. Written informed consent was obtained from eligible patients by research personnel fluent in Spanish (G.C., M.P.F.). The study took place from July 6 to October 16, 2021, including 1-day follow-ups. The primary end point was the feasibility of using the Google Translate translation application conversation mode to assess postoperative pain and nausea among Spanish-speaking patients in the postanesthesia care unit (PACU). Patients were introduced to the application during the preoperative interview, with Spanish-speaking research personnel (G.C., M.P.F.) explaining study goals and how the study would be conducted in the PACU. Preformulated questions were played for patients in the PACU through the application using an iPad tablet (Apple) held by the research coordinator (G.C., M.P.F.) at set intervals when nurses would typically evaluate symptoms. Patients responded with yes or no and gave numbers for pain and nausea ratings. Patient ethnicity was self-reported per institutional standard of care.

Use of the translation application conversation mode was considered feasible if at least 90.0% of patients were able to answer all 5 questions asked. Secondary end points are described in eMethods in the Supplement. We estimated that with a 30-patient sample size, the 95% CI for a feasibility rate of 90% was 73.5% to 97.9%. SAS statistical software version 9.4 (SAS Institute) was used for all analyses. Data were analyzed from October to November 2021.

## Results

Among 30 patients (median [IQR] age, 62 [53-80] years; 15 [50.0%] men) who were enrolled, all spoke only Spanish and self-identified as Hispanic (Table 1). The success rate during the entire PACU stay at evaluating postoperative pain and nausea every time was 76.7%. Separately, 80.0% and 83.3% of patients could be assessed using the application every time for pain or nausea, respectively. Most patients (83.3%) could communicate via the application on their first assessment attempt, and most (96.7%) were able to use the application successfully to answer all questions at least once during their PACU stay, with no patients needing standard institutional translation services.

**Table 1.** Patient Characteristics

Characteristic	Patients, No. (%) (N = 30)
Age, median (IQR), y	62 (53-80)
Sex	
Women	15 (50.0)
Men	15 (50.0)
Hispanic ethnicity	30 (100)
Nationality	
Bolivian	1 (3.3)
Chilean	1 (3.3)
Cuban	2 (6.7)
Dominican	1 (3.3)
Guatemalan	1 (3.3)
Mexican	21 (70.0)
Puerto Rican	1 (3.3)
Salvadorian	1 (3.3)
Uruguayan	1 (3.3)
ASA Score	
2	3 (10.0)
3	27(90.0)
Type of surgery	
Gastrointestinal	10 (33.3)
Thoracic	4 (13.3)
Orthopedic	2 (6.7)
Gynecologic	1 (3.3)
Breast Oncology	5 (16.7)
Urologic	7 (23.3)
Combined	1 (3.3)

Abbreviation: ASA, American Society of Anesthesiologists.

Most patients (73.3%) were satisfied or very satisfied with standard human translation services. Most patients (93.1%) were also satisfied or very satisfied with their pain and nausea management in the PACU, and 96.6% of patients were satisfied or very satisfied with the ability of the translation application to assess their symptoms (Table 2). One patient was unable to remember any details about the PACU experience.

**Table 2.** Summary of Outcomes

Outcome	Patients, No. (%) (N = 30)
**Primary end point**	
Able to use application at least once for	
Pain	
Yes	29 (96.7)
No	1 (3.3)
Nausea	
Yes	30 (100)
No	0
Pain and nausea	
Yes	29 (96.7)
No	1 (3.3)
Failed at least once overall for	
Pain	
Yes	2 (6.7)
No	28 (93.3)
Nausea	
Yes	1 (3.3)
No	29 (96.7)
Pain and nausea	
Yes	4 (13.3)
No	26 (86.7)
Did not ever fail	
Yes	23 (76.7)
No	7 (23.3)
First assessment attempt successful for	
Pain	
Yes	25 (83.3)
No	5 (16.7)
Nausea	
Yes	28 (93.3)
No	2 (6.7)
Pain and nausea	
Yes	25 (83.3)
No	5 (16.7)
**Secondary end point**	
Time in PACU, median (IQR), h	1.30 (0.45-1.55)
Postoperative pain present at first assessment in PACU	
Yes	25 (83.3)
No	5 (16.7)
Postoperative opioids required	
Yes	25 (83.3)
No	5 (16.7)
Postoperative opioid amount, MEDD, median (IQR)	9.00 (5.00-11.91)
Postoperative nonopioid analgesic	
Yes	12 (40.0)
No	18 (60.0)
Postoperative nausea present at first assessment in PACU	
Yes	8 (26.7)
No	22 (76.7)
Postoperative vomiting	
Yes	2 (6.7)
No	28 (93.3)
Postoperative antiemetics required	
Yes	6 (20.0)
No	24 (80.0)
Patient satisfaction with standard translation services	
Highly dissatisfied	0
Somewhat dissatisfied	2 (6.7)
Neutral	6 (20.0)
Somewhat Satisfied	9 (30.0)
Highly satisfied	13 (43.3)
Patient satisfaction with pain management in PACU	
Highly dissatisfied	0
Somewhat dissatisfied	0
Neutral	2 (6.9)
Somewhat satisfied	2 (6.9)
Highly satisfied	25 (86.2)
Patient satisfaction with nausea management in PACU	
Highly dissatisfied	0
Somewhat dissatisfied	0
Neutral	2 (6.9)
Somewhat satisfied	2 (6.9)
Highly satisfied	25 (86.2)
Patient satisfaction with translation application conversation mode ability to assess symptoms	
Highly dissatisfied	1 (3.4)
Somewhat dissatisfied	0
Neutral	0
Somewhat satisfied	4 (13.8)
Highly satisfied	24 (82.8)
Nurse satisfaction with quality of standard translation services	
Highly dissatisfied	0
Somewhat dissatisfied	0 (10.0)
Neutral	1 (3.3)
Somewhat satisfied	14 (46.7)
Highly satisfied	15 (50.0)
Nurse satisfaction with the immediate availability of standard translation services	
Highly dissatisfied	0
Somewhat dissatisfied	1 (3.3)
Neutral	0
Somewhat satisfied	22 (73.3)
Highly satisfied	7 (23.3)
Nurse satisfaction with speed translation application conversation mode could be used to assess patients	
Highly dissatisfied	0
Somewhat dissatisfied	2 (6.7)
Neutral	3 (10.0)
Somewhat satisfied	8 (26.7)
Highly satisfied	17 (56.7)
Nurse satisfaction with use of translation application conversation mode to assess patients	
Highly dissatisfied	0
Somewhat dissatisfied	3 (10.0)
Neutral	1 (3.3)
Somewhat satisfied	6 (20.0)
Highly satisfied	20 (66.7)

Abbreviations: MEDD, morphine equivalent daily dose; PACU, postanesthesia care unit.

Most nurses (96.7%) were satisfied or very satisfied with the quality and immediate availability of current institutional human translation services. Additionally, 83.3% and 86.7% of nurses were satisfied or very satisfied with the speed and ability that the translation application could be used for patient assessment, respectively (Table 2).

## Discussion

Disparities owing to race and ethnicity exist in postoperative symptom management across various settings^2^ and can be associated with poor clinical outcomes. In this cohort study, we report the successful use of a web-based electronic translating tool for assessment of postoperative symptoms among Spanish-speaking patients. Although Patil et al^3^ found that this translation application was suboptimal for comprehensive medical communication, the authors also found that the application was a useful adjunct tool when human translation services were unavailable. Other brief reports have documented the application as a potential perioperative communication tool.^4,5,6^ Limitations of our study included a small sample size and conduct of assessments in 1 language.

We observed that more than 90.0% of patients were able to communicate their pain and nausea using the translation application. Use of this technology could potentially be associated with decreased disparities in postoperative symptom assessment via improved patient-nursing communication.

## Notes

Supplement.
eMethods.
