# Supplementary material for: Use of Neural Machine Translation Software for Patients With Limited English Proficiency to Assess Postoperative Pain and Nausea
Source: JAMA Netw Open. 2022 Mar 8;5(3):e221485. doi: 10.1001/jamanetworkopen.2022.1485 (PMC8905382; doi:10.1001/jamanetworkopen.2022.1485)
Supplement: Supplement. — eMethods. [file jamanetwopen-e221485-s001.pdf]

Supplemental Online Content

Kapoor R, Corrales G, Flores MP, Feng L, Cata JP. Use of neural machine translation software for patients with limited English proficiency to assess postoperative pain and nausea. *JAMA Netw Open*. 2022;5(3):e221485. doi:10.1001/jamanetworkopen.2022.1485

eMethods.

This supplemental material has been provided by the authors to give readers additional information about their work.

© 2022 Kapoor R et al. *JAMA Network Open.*

eMethods.

Patients were eligible for the study if they met the following inclusion and exclusion criteria.

Inclusion Criteria: **1.** Ability to provide written informed consent, **2.** Patients between 18-80 years of age whose primary language is Spanish and will require translation services as part of standard care, **3.** American Society of Anesthesiologists (ASA) physical status 1-3, **4.** Are scheduled for surgery under general anesthesia and **5.** Ability to understand and complete all study questionnaires

Exclusion criteria: **1.** Emergency surgery**, 2.** American Society of Anesthesiologists physical status ≥ 4, **3.** Patients who do not require official translation services for consent purposes, **4.** Patients who have chronic pain and/or are taking opioids chronically (daily intake for more than a month before surgery), **5.** Patients who undergo craniotomy and **6.** Patients with a hearing impairment that prevents the ability to hear the recorded statements; and/or a cognitive impairment that would prevent them from completing post-operative assessments

Demographic data (i.e. age and gender), relevant medical history, type of surgery, and perioperative vital signs were collected from the electronic medical record. Postoperative pain and nausea scores were assessed by the PACU nurse. Research personnel played the validated assessment questions from the application. If clarification was needed on what was being asked after three consecutive attempts, the nurse could resort to official interpretation services as per standard of care. Patient responses to the nurses, who were non-Spanish speaking, were translated through Google Translate as well. If more than three attempts are needed for understanding the patient, the nurse could also resort to official interpretation services as per standard of care. Patients were assessed every fifteen minutes by the PACU nurse from the time the patient was deemed appropriate for verbal assessment by the nurse after arriving to the PACU until the nurse deemed the patient "ready for discharge" according to standard of care. The appropriateness for assessment was at the same time at which a nurse would traditionally go through official interpretation services to assess a non-English speaking patient as per standard of care. Data on time spent in PACU, amounts of pain/anti-emetics given, patient satisfaction

© 2022 Kapoor R et al. *JAMA Network Open.*

scores, and whether interpretation services were required will be collected on postoperative day #1.

Assessment of Pain and Nausea: Pain and nausea were assessed during the preoperative interview and again in the PACU as YES or NO. If YES, then, the intensity of both symptoms was evaluated using a verbal numeric reporting scale (0: no pain/nausea – 10: worst pain/nasuea possible). In the case of pain, if it was present, the location was also assessed. Pain and nausea assessments were conducted every 15 minutes from the time the patient was appropriate for assessment until the nurse deemed the patient "ready for discharge" according to standard of care. Throughout the patient's stay in PACU, until deemed "ready for discharge," the study coordinator or research fellow documented whether the patient understood what was being asked through Google Translate and whether the nurse understood the patient as well. If a patient failed to answer questions about the presence (YES/NO) of pain or nausea, their intensity (0-10) or location of pain after three consecutive attempts, or if a nurse failed to understand after three consecutive attempts, nurses could revert to institutional translation services as per standard of care. If this happened at any time, the research coordinator would stop conducting the study since official language services were or could be used from there onwards. An official institutional video translation device was kept at the patients' bedside, so that there were no delays in reverting to standard of care.

Questions for pain

Do you have pain? (Tienes dolor?)

If yes, then a second follow up question would be asked:

Can you rate your pain on a scale from 0-10? (Puedes valorar tu dolor de 0 a 10?)

Where 0 is feeling no pain and 10 is worst possible pain.

Where is your pain? (Donde te duele?)

Questions for nausea

Do you have nausea? (Tienes nauseas?)

If yes, then a second follow up question would be asked:

Can you rate your nausea on a scale from 0-10? (Puedes calificar sus nauseas de 0 a 10?)

Length of stay in PACU

© 2022 Kapoor R et al. *JAMA Network Open.*

The duration of time between patient arrival to PACU and ready for discharge according to standard of care was collected from the electronic medical record.

<u>Patient Satisfaction Scores:</u> Patient satisfaction scores from 1-5 were collected via a phone call or in-person visit through an interpreter prior to the surgery and then the day after surgery (POD #1). Scores were graded on the following scale: 1-Highly dissatisfied, 2-Somewhat dissatisfied, 3-Neutral, 4-Somewhat satisfied, 5-Highly satisfied

The following question was asked in Spanish by a native Spanish speaking study coordinator or research fellow or through official translation services prior to the surgery:

"How satisfied are you with the quality of medical translation services at our hospital?"

The following questions were asked in Spanish by a native Spanish speaking study coordinator or research fellow or through official translation services on POD#1:

"How satisfied were you with regards to your pain management in PACU?"

"How satisfied were you with regards to your nausea management in PACU?"

"How satisfied were you with the use of Google Translate to help assess your pain and nausea?"

<u>Nursing Satisfaction Scores:</u> Nursing satisfaction scores from 1-5 were collected at the time the patient was assigned to the specific PACU nurse and then again at the end of the patient's PACU stay by a native Spanish speaking study coordinator or research fellow or through official translation services. Scores were graded on the following scale: Scores were graded on the following scale: 1-Highly dissatisfied, 2-Somewhat dissatisfied, 3-Neutral, 4-Somewhat satisfied, 5-Highly satisfied

The following questions were asked by the research coordinator or research fellow to the nurse prior to the patient's arrival in PACU:

"How satisfied are you with the immediate availability of translation services at our hospital?"

"How satisfied are you with the quality of the current translation services at our hospital?"

The following questions will be asked by the research coordinator or research fellow to the nurse after the patient's discharge from the PACU:

"How satisfied are you with the speed with which you could use Google Translate to assess your patient's pain and nausea?"

© 2022 Kapoor R et al. *JAMA Network Open.*

“How satisfied were you with the ability of Google Translate to facilitate your assessment of pain and nausea for this patient?”

Statistical Considerations:

A total of 30 patients were enrolled for this pilot feasibility study. A patient needed to answer the following five questions: 1) the presence of pain (YES/NO); 2) the intensity of pain (0-10); 3) the location of pain; 4) the presence of nausea (YES/NO); and 5) the intensity of nausea (0-10). The primary endpoint was whether a patient was able to respond to the questions when asked using Google Translate. A patient was considered being able to use Google Translate if the patient could answer all the 5 questions without resorting to official translation services at least once during their PACU stay. The study was feasible if at least 90% patients were able to use Google Translate to answer all the 5 questions. When the sample size is 30, the exact 95% confidence interval (CI) for a feasibility rate of 90% is (73.5%, 97.9%).

© 2022 Kapoor R et al. *JAMA Network Open.*
